# Correlations between the metabolic profile and ^18^F-FDG-Positron Emission Tomography-Computed Tomography parameters reveal the complexity of the metabolic reprogramming within lung cancer patients

**DOI:** 10.1038/s41598-019-52667-8

**Published:** 2019-11-07

**Authors:** Karolien Vanhove, Michiel Thomeer, Elien Derveaux, Ziv Shkedy, Olajumoke Evangelina Owokotomo, Peter Adriaensens, Liesbet Mesotten

**Affiliations:** 1 Faculty of Medicine and Life Sciences, Hasselt University, Agoralaan building D, B-3590 Diepenbeek, Belgium; 2 Department of Respiratory Medicine, Ziekenhuis Oost Limburg, Schiepse Bos 6, B-3600 Genk, Belgium; 3 Institute for Biostatistics and statistical Bioinformatics, Hasselt University, Agoralaan Building D, B-3590 Diepenbeek, Belgium; 4 Applied and Analytical Chemistry, Institute for Materials Research, Hasselt University, Agoralaan Building D, B-3590 Diepenbeek, Belgium; 5 Department of Nuclear Medicine, Ziekenhuis Oost Limburg, Schiepse Bos 6, B-3600 Genk, Belgium

**Keywords:** Cancer, Oncology

## Abstract

Several studies have demonstrated that the metabolite composition of plasma may indicate the presence of lung cancer. The metabolism of cancer is characterized by an enhanced glucose uptake and glycolysis which is exploited by ^18^F-FDG positron emission tomography (PET) in the work-up and management of cancer. This study aims to explore relationships between ^1^H-NMR spectroscopy derived plasma metabolite concentrations and the uptake of labeled glucose (^18^F-FDG) in lung cancer tissue. PET parameters of interest are standard maximal uptake values (SUV_max_), total body metabolic active tumor volumes (MATV_WTB_) and total body total lesion glycolysis (TLG_WTB_) values. Patients with high values of these parameters have higher plasma concentrations of N-acetylated glycoproteins which suggest an upregulation of the hexosamines biosynthesis. High MATV_WTB_ and TLG_WTB_ values are associated with higher concentrations of glucose, glycerol, N-acetylated glycoproteins, threonine, aspartate and valine and lower levels of sphingomyelins and phosphatidylcholines appearing at the surface of lipoproteins. These higher concentrations of glucose and non-carbohydrate glucose precursors such as amino acids and glycerol suggests involvement of the gluconeogenesis pathway. The lower plasma concentration of those phospholipids points to a higher need for membrane synthesis. Our results indicate that the metabolic reprogramming in cancer is more complex than the initially described Warburg effect.

## Introduction

Metabolic adaptation in cancer cells was one of the first studied aspects of cancer. Otto Warburg discovered that, even in the presence of abundant oxygen, glycolysis leading to lactate via fermentation of pyruvate was often enhanced in cancer cells^1^. This phenomenon is known as the Warburg effect or, although somewhat confusing, “aerobic glycolysis”^2^. The high rates of glucose metabolism associated with the Warburg effect have been effectively exploited to facilitate tumor imaging by fluorodeoxyglucose-positron emission tomography (^18^F-FDG-PET)^3^. The radioactive tracer is taken up into malignant cells by upregulated glucose transporters (GLUT) and is subsequently trapped after phosphorylation by overexpressed hexokinase^4,5^. The most common parameter used to measure the uptake of ^18^F-FDG is the standardized uptake value (SUV) by positioning a region of interest (ROI) centrally within the tumor. There are two common ways of reporting SUV: the mean (SUV_mean_) or maximum SUV (SUV_max_) of all voxels in the ROI. In contrast with SUV_max_, the SUV_mean_ incorporates information of multiple voxels making it less sensitive to noise but more sensitive to ROI definition and subject to observer variability^6^.

The major disadvantage of SUV_max_ (the highest voxel value within the ROI) is the large degree of variability due to physical and biological sources of errors, as well as inconsistent and non-optimal image acquisition, processing and analysis^6^. In the study of cancer metabolism, we have to take into account that a single voxel ignores the extent of the metabolic changes^7^. Parameters that take this extent into account may contain more information about the disturbed glucose metabolism^8,9^. The metabolic active tumor volume (MATV), defined as the volume of hypermetabolic tissue with a SUV higher than a certain threshold, reflects the entire mass of the malignant tissue. The total lesion glycolysis (TLG), obtained by multiplying the MATV value with the mean SUV value of this volume, is a hybrid parameter that reflects not only volumetric information but includes also the intensity of the metabolic changes^9^.

Cancerous cells needs to activate specific metabolic pathways in order to develop into solid tumors and the concept of reprogramming of cancer metabolism is broader than the Warburg effect^10–12^. Indeed, cancer is not only characterized by enhanced glycolysis but also by upregulation of pathways emanating from glycolysis such as the pentose phosphate pathway, the hexosamines biosynthesis, the serine pathway and the one-carbon metabolism^13–16^. Furthermore, cancer cells take up large amounts of glutamine, which is critical for the generation of anti-oxidants to remove reactive oxygen species (ROS) and for the synthesis of nonessential amino acids, nucleotides and fatty acids^11,12,17–20^. Together, these pathways generate sufficient levels of cellular components to support cell proliferation. Metabolomics is defined as the “quantitative measurement of the dynamic multiparametric response of a living system to pathophysiological stimuli or genetic modification”^21^. Pathophysiological conditions such as cancer results in altered levels of metabolites or different metabolic profiles. Individual changes within the metabolome are useful for the identification of diagnostic and prognostic biomarkers, as well as for the identification of novel therapeutic targets and prediction of drug efficiency^22,23^. In contrast with other omics fields such as genomics and proteomics, metabolomics measures the true processes occurring within the patient, i.e. the phenotype. Therefore, the metabolome is the final downstream product of gene transcription and as consequence the metabolome is the closest to the phenotype of the biological system studied.

Although the study of altered metabolism in cancer cells is a relative new domain in oncology, several research groups were able to establish distinct metabolic profiles between cancer patients and healthy subjects using high resolution magic angle proton nuclear magnetic resonance (^1^H-NMR) spectroscopy^24–29^. In addition, several research groups were able to establish distinct metabolic plasma profiles between cancer patients and healthy subjects and between different cancer types^30–35^. Active efforts are ongoing in the search for metabolomics biomarkers that have relevance to lung cancer detection in biofluids such as blood and urine^24,34–40^. In general the principal metabolic alterations reported for lung cancer include changes in amino acid metabolism, choline phospholipid metabolism, glycolysis, one carbon-metabolism and lipid metabolism. The advantage of metabolic profiling of biofluids such as blood and urine is the potential to assess the complex interaction between tumor and host which is likely to play a critical role in defining prognosis and response to therapy. A recent study by our research group allowed the detection of lung cancer by metabolic phenotyping of plasma with ^1^H-NMR^34^.

The result of that study was the motivation to explore possible correlations between PET parameters and the plasma metabolic fingerprint. In contrast to what would be expected from the Warburg hypothesis, the authors revealed higher plasma concentrations of glucose in patients with lung cancer and assigned this to an increased gluconeogenesis in lung cancer patients. The aim of this paper is to explore possible relationships between metabolic imaging by ^18^F-FDG and plasma metabolite concentrations by ^1^H-NMR. This study may result in a deeper insight in the disturbed metabolism and guide us to the development of novel biomarkers and therapeutic agents for effective treatment.

## Material and Methods

### Subjects

In this retrospective study, lung cancer patients (N = 273) were prospectively enrolled in the NCT02024113 trial from March 2011 to June 2014 at the Limburg PET-Center (Hasselt, Belgium). In this trial, the investigators evaluated whether the metabolic profile of blood plasma of lung cancer patients allows the detection of lung cancer^34^. 34 individuals were excluded from the study based on overestimation risk of the MATVs due to incorporation of noncancerous regions nearby malignant lesions. In 13 subjects, tumors were considered as PET-negative (SUV_max_ < 2.5). 4 PET-CT scans were unusable due to technical defects. PET and patient characteristics (N = 222) are summarized in Table 1.

**Table 1:** Clinical and pathological characteristics of the patients.

Parameter	Median	Q1-Q3
SUV_max_	10.9	7.2–15.6
SUV_mean_	4.6	3.8–5.8
MATV_WTB_ (cm³)	48.1	9.7–170.8
TLG_WTB_ (cm³)	276.2	44.4–923.0
Glycemia (mg/dl)	101.5	92–114
BMI (kg/m²)	24.8	22.8–28.0
Age (years)	69	60–76
Parameter	Number (%)	
TNM stage		
I	58 (26.1%)	
II	26 (11.7%)	
III	78 (35.2%)	
IV	60 (27.0%)	
Histology		
Adenocarcinoma	82 (36.9%)	
Squamous cell	62 (27.9%)	
NOS	9 (4.1%)	
SCLC	33 (14.9%)	
No histology	26 (11.7%)	
Other	10 (4.5%)	
Smoking		
Active	113 (50.9%)	
Former	104 (46.8%)	
Never	5 (2.3%)	
Gender		
Female	70 (31.5%)	
Male	152 (68.5%)	

BMI: body mass index; MTV_WTB_: total metabolic active tumor volume; NOS: not otherwise specified; SCLC: small cell lung cancer; SUV: standardized uptake value; TLG_WTB_: total tumor lesion glycolysis; TNM, tumor node metastasis.

The original study was conducted in accordance with the ethical rules of the Helsinki Declaration and Good Clinical Practice and was approved by the ethics committees of Hasselt University and Ziekenhuis Oost-Limburg (ZOL, Genk). For this sub analysis a new informed consent was waived by these ethics committees.

### Blood sampling, sample preparation and NMR analysis

Fasting venous blood (10cc) was collected in lithium-heparin tubes and stored at 4 °C within 5 minutes. Samples were centrifuged at 1600 *g* for 15 min within 8 hours after collection. Plasma aliquots (500 µl) were transferred into cryovials and stored at −80 °C. The plasma samples were analyzed within 6 months after collection. The effect of storage duration on the metabolic profile has been evaluated by Louis *et al*. and Pinto *et al*.^41,42^. These authors concluded that the plasma is stable at −80 °C for a least ten months. After thawing, plasma aliquots were centrifuged at 13000 *g* for 4 minutes at 4 °C. Next, 200 µl of the supernatant was diluted with 600 µl deuterium oxide (D_2_O) that contained 0.3 µg/µl trimethylsilyl-2,2,3,3-tetradeuteropropionic acid (TSP) as chemical shift reference. Until ^1^H-NMR analysis samples were placed on ice.

After mixing and transfer into 5 mm NMR tubes, the samples were acclimatized to 21.2 °C during 7 min. The ^1^H-NMR spectra were recorded on an Inova 400 MHz (9.4 Tesla) spectrometer at 21.2 °C. Slightly transverse relaxation weighted (*T*_2_-weighted) spectra were acquired using the Carr–Purcell–Meiboom–Gill pulse sequence (total spin-echo time: 32 ms; interpulse delay: 0.1 ms), preceded by an initial preparation delay of 0.5 s and 3 s presaturation for water suppression. Other acquisition parameters were: spectral width of 6000 Hz, acquisition time of 1.1 s, 13k complex data points and 96 scans. Before Fourier-transformation, each free induction decay was zero-filled to 65k points, multiplied by a line broadening of 0.7 Hz, phased and referenced to TSP. The NMR spectrum was segmented into 110 fixed integrations regions (IRs), fixed on the basis of spiking plasma samples taken from a reference pool of a healthy volunteer with known metabolites^43^. For each metabolite, a different sample of the plasma pool was used. Signals of water and TSP were ignored. The spiking methodology allowed us to identify the metabolites appearing in 87 of these IRs. Including 23 additional IRs emanating from broader lipid signals and a few non-identified substances, the ^1^H-NMR spectrum could be divided into 110 well-defined IRs.

Subsequently, the spectra were baseline corrected and integrated. The resulting metabolic profile consists of 110 numerical integration values, i.e. the area under the peaks of these 110 IRs, representing the metabolite concentrations. By normalizing the integration values to the total integrated area, except water and TSP, relative concentrations were obtained. These are the variables for the statistical analysis. Several issues rationalize this spiking method: (i) it is preferred above peak assignments based on chemical shift values reported for different matrices and even non-human species, and (ii) in contrast to binning, the spiking method avoids that peaks are split into parts which might result in a loss of differentiating power^29,44,45^. A brief description of the information found in a ^1^H-NMR spectrum is given in the Supplemental Fig. S1.

### ^18^F-FDG PET-CT protocol

All images were acquired using a combined PET/CT scanner (GEMINI TF Big Bore, Philips). Patients fasted for at least six hours prior to the scan but were excluded if they had a capillary glucose ≥ 200 mg/dl. Image acquisition started one hour after administration of 3.75 MBq/kg ^18^F-FDG. After determination of the imaging field a low-dose CT of ±30 seconds (80–175 mAs, 120 kV), which ranged from the mid thighs to the base of the skull, was performed. The obtained CT images were reconstructed onto a 512–512 matrix. After the CT-scan, a PET-scan of 15 to 20 minutes which covers the same axial field, was performed. The emission time per bed position ranged from 1 to 2 minutes, depending on the body mass index (BMI) of the patient.

### Image analysis

**Figure 1:**
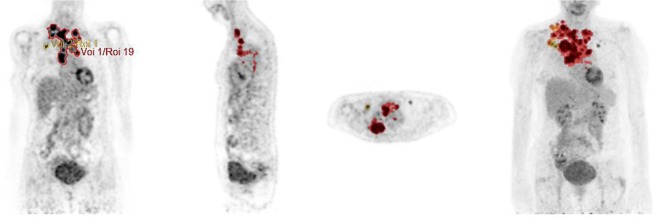
Visualization of the segmentation method used to quantify SUV_max_, SUV_mean_, MATV_WTB_ and TLG_WTB_. All lesions were evaluated separately. To calculate the SUV of a lesion, a region of interest was drawn (ROI) on the attenuation-corrected image. The computer calculates the maximum density in each ROI and reports these values as the SUV_max_. The software creates a 3D contour using voxels that are equal or greater as 2.5 and defines this volume as the metabolic active tumor volume (MATV). Subsequently, the average metabolic activity (SUV_mean_) of each MATV is calculated. The total lesion glycolysis (TLG) is calculated as MATV multiplied by its corresponding SUV_mean_. To obtain the whole body MATV and TLG of a patient, the values of all lesions were added.

Images were assessed using computer programmed analysis (Hermes Hybrid Viewer) in transverse, sagittal, and coronal planes (Fig. 1). Reports of a nuclear medicine physician were used as a reference when identifying the lesions. PET-CT parameters (SUV_max_, MATV and TLG) were obtained for the primary tumor, the involved lymph nodes and all the metastatic sites. MATV_WTB_ was defined as the total segmented volume of all hypermetabolic tissue i.e. with SUV ≥ 2.5. The TLG for a single tumor lesion is the product of its MATV and the SUV_mean_ for the lesion. TLG_WTB_ was calculated as the sum of the TLGs of all segmented tumors.

### Statistical analysis

The metabolic profile is defined as a set of 110 integration values (the variables for statistical analysis as described above) representing the relative metabolite concentrations, i.e. it consists of 110 numerical values that each represent the area under the peak(s) of an integrated region (IR) of the ^1^H-NMR spectrum. As described by Louis *et al*., 45 of these values were found to be significantly down- or upregulated in the plasma of lung cancer patients as compared to healthy controls and only these 45 regions are considered in this study^34^. Taking into account that a single metabolite can give rise to several signals in a ^1^H-NMR spectrum, the integration values of signals from a same metabolite are added. This resulted in a final data reduction up to 22 variables as shown in Tables 2 and 3. Both tables show the metabolites that are down or upregulated in the plasma of lung cancer patients as compared to healthy controls. The shape of the histograms and the Kolmogorov-Smirnov statistic were used to assess the distribution of the data. PET parameters were dichotomized by their median values and patients were divided into a low-value and high-value group for each PET parameter (SUV_max_, MATV and TLG).

**Table 2:** Mann-Whitney test between the two groups formed on the basis of the median values of the PET parameters. The reported values are the effect size. A negative effect size is consistent with a higher concentration of the metabolite in the high-value group.

Metabolite	IR (ppm)	SUV_max_	P(corrected)	MATV_WTB_	P(corrected)	TLG_WTB_	
Glucose	5.2752–5.2526 4.6940–4.6620 3.9590–3.8330 3.8330–3.8100 3.8100–3.7956 3.7956–3.7820 3.7820–3.7550 3.7550–3.7390 3.7390–3.7141 3.5649–3.5510 3.5510–3.5360 3.5360–3.3980	−5.5	1	−**19.2**	*0.03*	−**22.7**	*0.01*
NAG	2.1230–1.9720	−**2.7**	*0.05*	−**2.7**	*0.005*	−**2.8**	*0.005*
Glycerol	3.7141–3.6680 3.6680–3.6500 3.6500–3.6376 3.5914–3.5649	−1.2	0.5	−**2.3**	*0.005*	−**2.4**	*0.005*
Threonine	3.6240–3.6097 3.6097–3.5914	−0.5	0.2	−**1.1**	*0.005*	−**1.1**	*0.005*
Valine	3.6376–3.6240 1.0220–1.0020	−0.5	0.08	−**0.7**	*0.005*	−**0.8**	*0.005*
Leucine	1.8060–1.6860 1.0020–0.9860	−0.4	0.7	−0.9	0.1	−0.9	0.07
α-ketoglutarate or lysine	3.0640–2.9950	−0.4	0.9	0.2	0.51	−0.05	0.9
Phospholipids** -C**H**_2_-C = O or -C**H**_2_-CH = CH-	2.3040–2.2915 2.2915–2.2690 2.2690–2.2300	−0.4	0.5	0.3	0.6	0.5	0.6
Asparagine	2.9950–2.8860	−0.4	0.9	0.6	0.4	0.3	0.7
Phospholipids** = CH-C**H**_2_-CH =	2.8550–2.7500	−0.3	0.5	0	0.51	0	0.5
Aspartate	2.7360–2.6600	−0.2	0.4	−**0.4**	*0.005*	−**0.4**	*0.005*
Phospholipids** -C**H**_2_-CH_2_-C = O or -C**H**_2_-CH_2_-CH = CH-	1.6860–1.5600	−0.03	1	0.7	0.2	0.7	0.1
Tyrosine	3.2186–3.1930	−0.02	0.6	0.03	0.7	0.02	0.9
Amino-acid group*	3.9810–3.9590	0	1	−**0.4**	*0.03*	−0.3	0.07
Citrate	2.5960–2.5340	0.1	0.5	0.08	0.5	0.06	0.6
Glutamine	2.4920–2.4500 2.1970–2.1230	0.3	0.2	0.2	0.5	0.04	0.5
β-hydroxybutyrate	1.2458–1.2180	0.3	0.5	−0.2	0.51	−0.2	0.6
Alanine	1.5400–1.4900	0.7	0.5	0.7	0.2	0.7	0.2
Phospholipids** -C**H** = C**H**-	5.4300–5.2752	1.2	1	1.8	0.1	**2.1**	*0.04*
Phospholipids**CH_3_-(C**H**_2_)_n_ and C**H**_3_-(CH_2_)_n_	1.3450–1.2458 0.9660–0.8000	1.4	1	14.0	0.08	**15.8**	*0.04*
Lactate	1.4200–1.3740 1.3740–1.3450	2.6	1	4.3	0.1	4.3	0.07
Phospholipids** N^+^(C**H**_3_)_3_ of SM and PC in lipoproteins	3.3230–3.2186	3.8	0.06	**4.4**	*0.03*	**4.4**	*0.02*

MATV_WTB_: total metabolic active tumor volume; NAG: N-acetylated glycoproteins; PC, phosphatidylcholines; SM: sphingomyelins; SUV: standardized uptake value; TLG_WTB_: total tumor lesion glycolysis. *This amino-acid group consists of asparagine, histidine, serine and tyrosine. **Signals of phospholipids at the surface of lipoproteins. Significant effect sizes are marked in bold and corresponding significant P- values in *italic*.

**Table 3:** Correlations between the quantitative PET parameters and the relative metabolite concentrations (from ^1^H-NMR) for lung cancer patients.

Metabolite	SUV_max_		MATV_WTB_		TLG_WTB_	
	R	P (corrected)	R	P (corrected)	R	P (corrected)
Threonine	**0.25**	*0.01*	**0.44**	*0.003*	**0.42**	*0.003*
NAG	**0.25**	*0.01*	**0.36**	*0.003*	**0.36**	*0.003*
Valine	**0.25**	*0.01*	**0.34**	*0.003*	**0.34**	*0.003*
Aspartate	**0.22**	*0.01*	**0.29**	*0.001*	**0.29**	*0.003*
Glycerol	**0.20**	*0.01*	**0.35**	*0.003*	**0.34**	*0.003*
Leucine	0.11	0.33	**0.19**	*0.01*	**0.19**	*0.01*
Glucose	0.10	0.38	**0.24**	*0.003*	**0.23**	*0.003*
Amino-acid group*	0.08	0.48	**0.28**	*0.003*	**0.26**	*0.003*
α-ketoglutarate or lysine	0.06	0.48	−0.003	0.96	−0.002	0.98
Tyrosine	0.06	0.50	−0.04	0.69	−0.03	0.82
Asparagnine	0.04	0.66	−0.06	0.46	−0.06	0.51
Phospholipids** = CH-C**H**_2_-CH =	0.02	0.86	−0.08	0.32	−0.07	0.39
Phospholipids** -C**H**_2_-C = O or -C**H**_2_-CH = CH-	0.01	0.94	−0.07	0.38	−0.06	0.48
β-hydroxybutyrate	0.00	0.94	0.05	0.96	0.01	0.96
Citrate	−0.06	0.48	−0.09	0.29	−0.09	0.30
Phospholipids** -C**H**_2_-CH_2_-C = O or -C**H**_2_-CH_2_-CH = CH-	−0.06	0.48	−0.12	0.13	−0.12	0.13
Glutamine	−0.07	0.48	−0.01	0.96	−0.02	0.903
Lactate	−0.07	0.48	−**0.16**	*0.04*	−**0.15**	*0.05*
Alanine	−0.08	0.48	−0.1	0.23	−0.10	0.21
Phospholipids** CH_3_-(C**H**_2_)_n_ and C**H**_3_-(CH_2_)_n_	−0.08	0.48	−**0.21**	*0.003*	−**0.20**	*0.01*
Phospholipids** -C**H** = C**H**-	−0.09	0.46	−0.18	0.14	−**0.17**	*0.02*
Phospholipids** N^+^(C**H**_3_)_3_ of SM and PC in lipoproteins	−**0.18**	*0.03*	−**0.21**	*0.005*	−**0.22**	*0.003*

Metabolites measured in the plasma of lung cancer patients based on the spiking experiment of Louis *et al*. MATV_WTB_: total  metabolic active tumor volume; NAG: N-acetylated glycoproteins; PC, phosphatidylcholines; SM: sphingomyelins; TLG_WTB_: total lesion glycolysis. Significant correlations (after FDR correction) are marked in italic and significant correlation coefficients in bold. *This amino-acid group consists of asparagine, histidine, serine and tyrosine. **Signals of phospholipids at the surface of lipoproteins.

The non-parametric Mann-Whitney test was used to detect significant statistical differences between the two groups using an overall significance level of 5%. To avoid an inflated type I error by multiple testing we applied the false discovery rate (FDR) method of Benjamini-Hochberg^46^. The effect size of each metabolite was calculated as the difference between the median value in the low-value and high-value group. To investigate correlations between the plasma metabolite concentrations and PET parameters, the Spearman correlation coefficient was used and a FDR correction was also applied. We evaluated the differences in metabolite concentrations between patients with high and low PET-parameters within each substage using the Mann-Whitney test with FDR correction.

All tests were performed using SPSS Statistics (IBM SPSS statistics, version 24.0; SPSS Inc.).

Multivariate statistics was performed using SIMCA-P+ (Version 14.0, Umetrics, Umea, Sweden). Identification of clusters within the dataset was accomplished via an unsupervised principal component analysis (PCA) by which outliers were detected on the basis of a Hotelling’s T2 range and a distance to model plot. Orthogonal Partial Least Squares Discriminant Analysis (OPLS-DA) was performed to construct a classification model to discriminate between patients with high and low values of the PET-parameters^47^. Models were compared on the basis of the total amount of explained variation (R^2^X(cum) and R^2^Y(cum)), predictive ability (Q^2^(cum)) and the levels of sensitivity (the percentage of patients that are actually classified as patients with high values of the parameter of interest) and the specificity (the percentage of patients that are actually classified as patients with low values of the parameter of interest). NMR spectra were normalized in order to account for concentration differences between plasma samples^48^. To avoid that the most abundant metabolites would dominate the resulting statistical models, NMR integration values were subjected to mean centering and Pareto scaling^49^.

## Results

As the Kolmogorov-Smirnov statistic and the histograms revealed that the data are not normally distributed, we used non-parametric tests to study correlations and differences between groups. Patients (N = 222) were divided into a low-value and high-value group on the basis of their median values of one of the following PET parameters: SUV_max_ (10.9; 7.2–15.6), MATV_WTB_ (48.1 cm³; 9.7–170.8 cm³) and TLG_WTB_ (276.2 cm³; 44.4–923.0 cm³). Metabolites of which the relative plasma concentration is significantly different between the groups are summarized in Table 2. A negative effect size is consistent with a higher value of the metabolite in the high-value group. Patients with a SUV_max_ ≥ 10.9 have higher plasma concentrations of N-acetylated glycoproteins (NAGs). Blood plasma of patients with MATV_WTB_ ≥ 48.1 cm³ and TLG_WTB_ ≥ 276.2 cm³ is characterized by higher concentrations of glucose, glycerol, NAG, threonine, aspartate and valine, but lower levels of sphingomyelin (SM) and phosphatidylcholine (PC). As mentioned in Table 2, the signal of a choline head group, N^+^(CH_3_)_3_ at 3.32–3.22 ppm, reflects the specific phospholipids SM and PC at the surface of lipoproteins. The other assigned lipid signals in Table 2 arise, next to SM and PC, also from other phospholipids in lipoproteins such as e.g. phosphatidylserine or phosphatidylethanolamine, since these signals account for structure features generally found in phospholipids. In general, lower concentrations of phospholipids are found in the patient group with TLG_WTB_ ≥ 276.2 cm³. In addition, patients with a MATV_WTB_ ≥ 48.1 cm³ have higher plasma concentrations of the amino-acids asparagine, histidine, serine and tyrosine.

Significant univariate correlations between PET-values and plasma metabolite concentrations are shown in Table 3. The strongest correlation was found between the amino acid threonine and the volumetric parameters MATV_WTB_ (R = 0.44) and TLG_WTB_ (R = 0.42).

**Figure 2:**
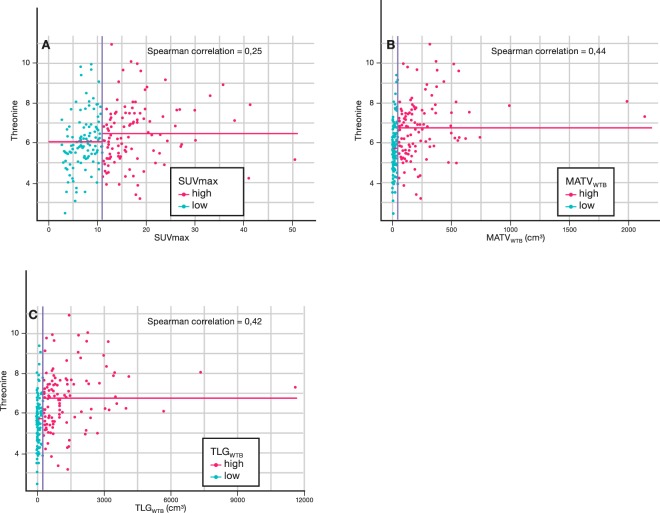
Discrimination and correlation of median-based PET-parameters. The vertical blue line is the median value of the PET parameter of interest. The horizontal pink lines are the median values of the metabolite of interest (here: threonine) in the two groups. The difference between the two horizontal pink lines is the effect size. The blue and pink dots represent the lung cancer patients of the low and high value groups, respectively.

Figure 2 shows the scatterplots of threonine versus SUV_max_, MATV_WTB_ and TLG_WTB_ (the horizontal lines represent the median threonine values in the two groups and the difference is the fold change) and reveals that, although the correlation between threonine and the PET parameters is rather weak, the fold change of especially MATV_WTB_ and TLG_WTB_ allows discrimination between the groups.

As patients with a higher stage of disease are likely to have higher values of MATV_WTB_ and TLG_WTB_ we evaluated the differences in metabolite concentrations between patients with high and low PET-parameters within each substage using the Mann-Whitney test (Table S1). In general, if a metabolite is significant between the low and high PET parameter group for a specific factor this means that the factor influences the distribution of the metabolites across the high versus low group and thus that the metabolic parameter may act as a confounder thereby influencing our interpretation. Concerning stage, significant discriminating metabolites were found between MATV_WTB_/TLG_WTB_ and substages (Table S1). The same workflow was performed to evaluate the role of gender and the histological subtype.

**Figure 3:**
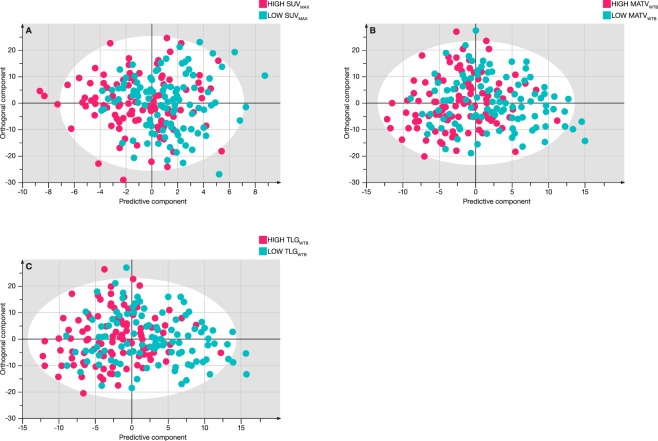
OPLS-DA score plots for the lung cancer patients after splitting in two groups on the basis of the median value of SUV_max_ (**A**), MATV_WTB_ (**B**) and TLG_WTB_ (**C**). Blue: low value, Pink: high value.

Although the number of patients of this study is still rather limited, multivariate OPLS-DA statistics was evaluated to discriminate between the low and high PET value groups on the basis of the metabolite concentrations that are contained in the metabolic phenotype. Table 4 shows the characteristics of the constructed models which are still relatively poor as also can be observed in the OPLS-DA plots of Fig. 3.

**Table 4:** Characteristics of the OPLS-DA models.

	R^2^X (cum)	R^2^Y (cum)	Q^2^ (cum)	Sens (%)	Spec (%)	MCE (%)
SUVmax high/low	0.88	0.144	0.07	65	60	37
MATV_WTB_ high /low	0.77	0.19	0.16	65	71	31
TLG_WTB_ high/low	0.77	0.20	0.16	66	72	31

MATV_WTB_: metabolic active tumor volume; SUV: standardized uptake value; TLG_WTB_ total lesion glycolysis; R^2^X: amount of variation within groups; R^2^Y: amount of variation between groups; Q^2^: predictive ability determined by internal cross-validation. Sens: sensitivity; Spec: specificity; MCE: misclassification error.

### Ethics approval and consent to participate

Sub analysis of NCT02024113 study where all the participants signed an informed consent. For this sub analysis a new informed consent was waived by the ethics committee. All procedures were in accordance with the ethical standards of the institutional research committee (Ziekenhuis Oost-Limburg Genk/Hasselt University) and with the 1964 Helsinki declaration and its later amendments.

## Discussion

Notwithstanding that the first discovery of metabolic changes in cancer occurred almost a century ago, altered metabolism has been recently acknowledged as a key hallmark of cancer^50^. As a consequence, metabolism-focused cancer research has received renewed attention^51–53^.

Recently, Louis *et al*. demonstrated that metabolic phenotyping of human plasma with ^1^H-NMR allows to detect lung cancer^34^. In contrast to what would be expected from the Warburg hypothesis, the plasma of lung cancer patients was shown to be characterized by an increased level of glucose. This higher level was assigned to an increased gluconeogenesis in normal cells to overcome the higher glucose uptake by malignant tissue. In contrast with glycolysis, gluconeogenesis is an anabolic pathway that requires non-carbohydrate precursors such as amino acids, lactate and glycerol for the generation of glucose.

These precursors arise from catabolic pathways such as lipolysis and proteolysis, or in case of amino acids, also by an enhanced dietary uptake. Accordingly, patients with a low uptake of ^18^F-FDG may not only exhibit lower concentrations of glucogenic precursors in their plasma but also use other pathways to sustain their growth and proliferation. This potential difference between patients with low and high ^18^F-FDG uptake was the stimulus to explore a possible relationship between the uptake of labeled glucose and the metabolic profile in plasma of lung cancer patients.

As can be seen from statistical analyzes in Tables 2 and 3, patients with a high maximal glucose uptake (SUV_max_), a large metabolic active tumor volume (MATV_WTB_) or a high glycolytic burden (TLG_WTB_) have a significant increase in the plasma concentration of N-acetylated glycoproteins or NAGs. The elevated glycolysis in lung cancer cell results in a higher production of fructose-6-phosphate, a metabolite that can branch of the glycolysis to enter the hexosamine biosynthesis pathway (HBP) where it becomes converted into intermediate products like UDP-N-acetylglucosamine that are critical for post-translational modifications of proteins, such as protein glycosylation to NAGs^54^.

These glycoproteins play a role in the regulation of growth, differentiation and metastasis^54^. NAGs are secreted from lung cancer cells into surrounding biological fluids such as plasma, explaining the higher levels when the glycolytic activity increases^55^. These findings further suggest a higher concentration of NAGs in glycolytic lung cancer tissue which was also demonstrated by the group of Higashi^56^. The concentrations of other metabolites were not significantly different for patients with a low or high ^18^F-FDG maximal glucose uptake (SUV_max_) as shown in Table 2. This is not completely unexpected since SUV_max_ values are subject to quite some variability^6,57^.

Table 2 clearly shows that the concentration of several other metabolites can discriminate between patients with a small and large metabolic active tumor volume (i.e. MATV_WTB_ ≥ 48.1 cm³) or low and high glycolytic burden (i.e. TLG_WTB_ ≥ 276.2 cm³). This result is somewhat expected since both volumetric parameters as well as blood plasma contain whole-body biological information.

Patients with large active tumor volumes and high glycolytic burden have significantly higher plasma concentrations of glucose, glycerol, NAGs, threonine, aspartate, valine on one hand but lower concentrations of SM and PC on the other hand (Table 2). Notwithstanding that both PET parameters correlate most strongly with threonine as shown in Table 3, the correlation remains modest. This means that the plasma level of threonine can be used to differentiate between the groups, but not to predict the values of these PET parameters. Contrary to what would be expected on the basis of the Warburg effect in cancer cell tissue, our findings indicate that the elevated glycolysis is associated with an increase of the plasma glucose. This higher plasma glucose in lung cancer patients has been previously described by Louis *et al*. and Chen *et al*.^34,58^. Louis *et al*. assigned the higher glucose concentration to a compensatory glycogenolysis and gluconeogenesis. More concrete, in response to the Warburg effect, glycogen stored in the liver and skeletal muscles is released resulting in depleted glycogen stores^59^. After depletion of the hepatic glycogen stores, glucose is formed in liver cells from non-carbohydrate precursors such as lactate, glycerol, and amino acids. As a result, the metabolic profile of plasma of lung cancer patients not only reflects the locally disturbed cancer cell metabolism but also the inherent nature of the normal body cells to supply all tissues with metabolic building blocks and fuel to function properly (homeostasis). This explains that higher concentrations of the glucogenic amino acids (aspartate, threonine, valine) are needed to keep the glucose levels intact in patients having a large MATV_WTB_ and/or high TLG_WTB_ (Table 2). The degradation of muscle proteins is an important source of these glucogenic amino acids^60,61^. Another homeostatic phenomenon, i.e. lipolysis of adipose tissue, results in the liberation of glycerol and free fatty acids. Besides for lipid synthesis, glycerol is another non-carbohydrate glucogenic substrate for glucose formation. Besides for the synthesis of triglycerides, fatty acids are key components of sphingomyelins (SM) and phosphatidylcholines (PC) needed for the formation of cell membranes of the fast growing malignant cells, explaining the lower concentration of SM and PC in the plasma of lung cancer patients^62,63^. More specifically, plasma phospholipids with ^1^H-NMR visibility like these cholinated SMs and PCs do not appear as individual molecules, but rather at the surface of lipoproteins^64^. In general, lipoproteins are classified in distinct classes based on their density, size and composition of core and surface components. While larger, (very) low-density lipoproteins ((V)LDL) contain a high concentration of triglycerides and cholesterol esters in their hydrophobic core, the concentration of phospholipids is significantly higher in smaller high-density lipoproteins (HDL)^65^. Therefore, it can be deducted that the detected signals of SM and PC mainly arise from those phospholipids appearing in HDL. Another argument to support this rationale can be found in the difference in rotational mobility and thus T2 relaxation decay time of the distinct lipoproteins. Due to their shorter T2 relaxation decay time, more signal of the larger LDL than of the smaller HDL will be suppressed by the CMPG filter used in our NMR measuring protocol. In line with the higher need of cell membrane formation, uptake of lipoproteins such as HDL is also often used by malignant cells. This is supported by the observation that solid tumors display an increased uptake of lipoproteins compared with healthy tissues^66–69^. Differences in metabolite concentrations between patients with high and low PET-parameters were evaluated between male and female and between histological- and stage subgroups as demonstrated in Table S1 in the supplementary information. Concerning stage, significant discriminating metabolites were found between the MATV and substages (Table S1). More specifically, β-hydroxybutyrate, NAGs, glycerol, threonine and glucose seem related with stage II and IV. Similar results were obtained between TLG and stage II, III and IV except for β-hydroxybutyrate and threonine in stage III. As β-hydroxybutyrate is not retained as a significant metabolite in Table 2 of our study, we controvert its importance. Furthermore, we controvert this potential confounding as there is no biological explanation since similar results were obtained in early (stage II) and advanced (stage IV) disease. In addition, the analysis in this study was performed on patients that were recruited in the NCT02024113-trial where no differences were seen in the metabolic profiles between the lung cancer stages^34^. In this trial orthogonal projections to latent structures - discriminant analysis (OPLS-DA) statistics were used to train a classification model in discriminating between patients with lung cancer and controls on the basis of data input from their metabolic phenotype. In a next step, whether tumor stages can be discriminated on the basis of the metabolic profile was evaluated. In an attempt to discriminate between patients with stage I and stage IV, a model was trained that correctly classified 79% of the patients with early stage and 52% of the patients with metastatic stage. However, the predictive ability (Q²) of the model was very low (Q² 0.06). Potential confounding was detected between the PET parameters and gender and between the PET parameters and histologic subtypes. Concerning histology, the squamous histology may have affected the interpretation of our results. However, the association between gender and histology were also evaluated in the NCT02024113-trial and no significant differences were detected between gender and histological subtypes. In this trial, an OPLS-DA model was trained to discriminate between the most common histological subtypes, i.e. adenocarcinoma and squamous carcinomas. The resulting model classified 81% of the adenocarcinomas correctly, but only 38% of the squamous carcinoma. However, in analogy with stage, the predictive ability Q² was poor (Q² 0.04).

Despite the significant differences between patients with a large and small MATV_WTB_ and between patients with a high and low TLG_WTB_, a multivariate OPLS-DA approach cannot significantly distinguish between these patient groups. Table 4 demonstrates the low predictive abilities (Q^2^ < 0,2), small variations between the groups (R^2^Y < 0,2), relative low sensitivities and specificities, and high misclassification errors. Although a strong OPLS-DA model could be constructed to discriminate between cancer patients and controls by Louis *et al*., no further discrimination seems possible in this study population between the patients having low versus high values for the volumetric PET parameters (MATV_WTB_ and TLG_WTB_). This also confirms the finding of Louis *et al*., in that metabolic phenotyping is not able to discriminate further between clinical stages in this study population.

Using the plasma metabolic phenotype we were able to identify distinct pathways that discriminates between low and high ^18^F-FDG uptake. The neoglucogenic pathway, the hexosamine biosynthesis pathway and the plasma membrane synthesis are upregulated in patients with intense glycolysis.

## Conclusion

The focus of this study was to investigate relationships between the plasma metabolite concentrations obtained by ^1^H-NMR spectroscopy and the glycolytic activity measured by PET-CT. It could be demonstrated by NMR metabolomics that a larger metabolic active tumor volume and/or a higher glycolytic burden seems to be associated with a higher glucose plasma concentration, indicative for an increased gluconeogenesis in this group of lung cancer patients. This higher ^18^F-FDG uptake also corresponds with higher concentrations (and need) of non-carbohydrate glucose precursors such as glycerol and glucogenic amino acids for which degradation of adipose tissue and muscle proteins are important sources. The higher plasma concentration of NAGs is in relation with an upregulated hexosamine biosynthesis, a pathway emanating from glycolysis. The reduced concentration of lipoproteins (mainly HDL) reflected by the lower concentration of phosphatidylcholines, sphingomyelins and other phospholipids in lipoproteins reflects the enhanced synthesis of plasma membranes for the fast growing cancer cells. These results confirm current knowledge that the metabolic reprogramming in cancer goes much wider than described by the Warburg effect.

## Supplementary information

Supplementary information

## Data Availability

Please contact author for data requests.
