# Supplementary material for: Correlations between the metabolic profile and 18F-FDG-Positron Emission Tomography-Computed Tomography parameters reveal the complexity of the metabolic reprogramming within lung cancer patients
Source: Sci Rep. 2019 Nov 7;9:16212. doi: 10.1038/s41598-019-52667-8 (PMC6838313; doi:10.1038/s41598-019-52667-8)
Supplement: Supplementary file 1 — Supplementary information [file 41598_2019_52667_MOESM1_ESM.docx]

**Correlations between the metabolic profile and ^18^F-FDG-Positron Emission Tomography-Computed Tomography parameters reveal the complexity of the metabolic reprogramming within lung cancer patients.**

Karolien Vanhove^1^, Michiel Thomeer^1,2^, Elien Derveaux^1^, Ziv Shkedy^3^, Olajumoke Evangelina Owokotomo^3^, Peter Adriaensens^4,*^ and Liesbet Mesotten^1,5^

**SUPPLEMENTARY TABLE S1**

| Significant differences in metabolite concentrations between patients with high and low PET-parameters within stage | | | | | | | | | | | |
| --- | --- | --- | --- | --- | --- | --- | --- | --- | --- | --- | --- |
|  | | Stage I | | | Stage II | | Stage III | | | Stage IV | |
| SUV_max_ | | Glucose | | | Glucose  Glycerol  NAG | |  | | |  | |
| MATV | |  | | | β-hydroxybutyrate  NAG  Glycerol  Threonine  Glucose | |  | | | β-hydroxybutyrate  NAG  Glycerol  Threonine  Glucose | |
| TLG | |  | | | β-hydroxybutyrate  NAG  Glycerol  Threonine  Glucose | | NAG  Glycerol  Glucose | | | β-hydroxybutyrate  NAG  Glycerol  Threonine  Glucose | |
| Significant differences in metabolite concentrations between patients with high and low PET-parameters within gender | | | | | | | | | | | |
|  | | | | Male | | | | | Female | | |
| SUV_max_ | | | |  | | | | |  | | |
| MATV | | | | β-hydroxybutyrate  Glucose  NAG  Glycerol  Threonine | | | | | Glucose  NAG  Glycerol | | |
| TLG | | | | β-hydroxybutyrate  Glucose  NAG  Glycerol  Threonine | | | | | Glucose  Glycerol | | |
| Significant differences in metabolite concentrations between patients with high and low PET-parameters within histological subgroups | | | | | | | | | | | |
|  | Unknown | | NOS | | | SCLC | | Squamous | | | Adenocarcinoma |
| SUV_max_ |  | |  | | |  | |  | | |  |
| MATV |  | |  | | |  | | β-hydroxybutyrate  Glucose  NAG  Glycerol | | |  |
| TLG |  | |  | | |  | | β-hydroxybutyrate  Glucose  NAG  Glycerol | | |  |

MATV_WTB_: total metabolic tumor volume; NAG: N-acetylated glycoproteins; SUV: standardized uptake value; TLG_WTB_: total tumor lesion glycolysis.

**SUPPLEMENTARY FIGURE 1**

**
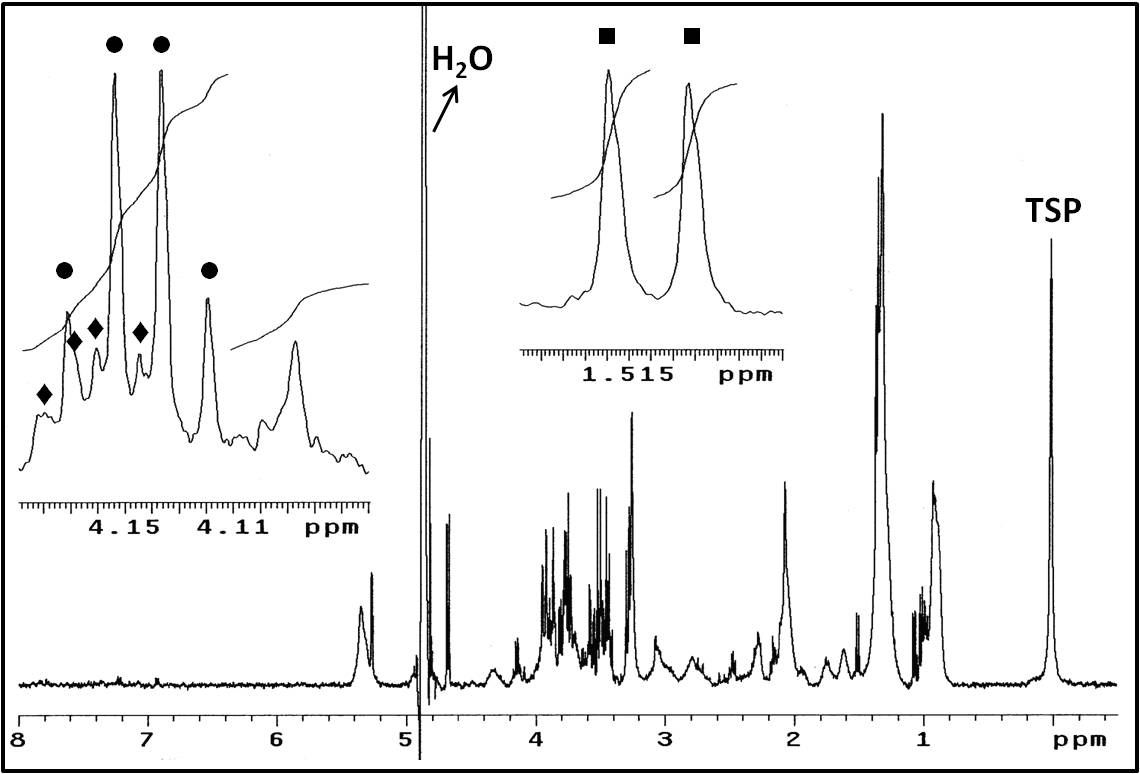
**

Figure S1 : Figure S1 shows a typical proton NMR spectrum of human plasma, with four illustrating integration regions (IRs) in the two insets. In this study, NMR spectra were divided into 110 integration regions of which the normalized integration values are the variables for multivariate statistics. Note that the 110 IRs do represent less than 110 metabolites since most metabolites have protons in different chemical environments and so give rise to more than one signal in the 1H-NMR spectrum. The inset at the right shows the integration regions of the alanine doublet lines (■) between 1.531.51 ppm (IR 82) and 1.51-1.49 ppm (IR 83). The doublet arises from the methyl group protons which are J-coupled to the Cα methine proton with a scalar coupling constant of J=7.2 Hz. The left inset shows the integration regions between 4.19-4.11 ppm (IR 16) and 4.11-4.06 ppm (IR 17). The IR 16 consists of the quadruplet lines (●) of lactate (CH_3_CHOHCOOH; the quadruplet results from the methine proton which is J-coupled to the three protons of the vicinal methyl group with a coupling constant of J=6.9 Hz), which are superimposed on the double doublet lines (♦) of the Hα proton of proline (J=8.7 and 6.0 Hz). The IR 17 is composed of signals arising from protons of creatinine, fructose, inositol and tryptophan.
